# Maize Genomes to Fields: 2014 and 2015 field season genotype, phenotype, environment, and inbred ear image datasets

**DOI:** 10.1186/s13104-018-3508-1

**Published:** 2018-07-09

**Authors:** Naser AlKhalifah, Darwin A. Campbell, Celeste M. Falcon, Jack M. Gardiner, Nathan D. Miller, Maria Cinta Romay, Ramona Walls, Renee Walton, Cheng-Ting Yeh, Martin Bohn, Jessica Bubert, Edward S. Buckler, Ignacio Ciampitti, Sherry Flint-Garcia, Michael A. Gore, Christopher Graham, Candice Hirsch, James B. Holland, David Hooker, Shawn Kaeppler, Joseph Knoll, Nick Lauter, Elizabeth C. Lee, Aaron Lorenz, Jonathan P. Lynch, Stephen P. Moose, Seth C. Murray, Rebecca Nelson, Torbert Rocheford, Oscar Rodriguez, James C. Schnable, Brian Scully, Margaret Smith, Nathan Springer, Peter Thomison, Mitchell Tuinstra, Randall J. Wisser, Wenwei Xu, David Ertl, Patrick S. Schnable, Natalia De Leon, Edgar P. Spalding, Jode Edwards, Carolyn J. Lawrence-Dill

**Affiliations:** 1 Iowa State University, Ames, IA 50011 USA; 2 University of Wisconsin, Madison, WI 53706 USA; 3 Cornell University, Ithaca, NY 14853 USA; 4 University of Arizona, Tucson, AZ 85721 USA; 5 University of Illinois at Urbana-Champaign, Urbana, IL 61801 USA; 6 USDA-ARS, Beltsville, MD USA; 7 Kansas State University, Manhattan, KS 66502 USA; 8 University of Missouri, Columbia, MO 65211 USA; 9 South Dakota State University, Rapid City, SD 57702 USA; 10 University of Minnesota, St. Paul, MN 55108 USA; 11 North Carolina State University, Raleigh, NC 27695 USA; 12 University of Guelph, Ridgetown, ON Canada; 13 University of Guelph, Guelph, ON Canada; 14 University of Nebraska, Lincoln, NE 68583 USA; 15 Pennsylvania State University, University Park, PA 16802 USA; 16 Texas A&M University, College Station, TX 77843 USA; 17 Purdue University, West Lafayette, IN 47907 USA; 18 University of Florida, Gainesville, FL 32611 USA; 19 Ohio State University, Columbus, OH 43210 USA; 20 University of Delaware, Newark, DE 19716 USA; 21 Texas A&M AgriLife Research, Lubbock, TX 79403 USA; 22 Iowa Corn Growers Association, Johnston, IA 50131 USA; 23 Present Address: University of Wisconsin, Madison, WI 53706 USA; 24 Present Address: University of Missouri, Columbia, MO 65211 USA; 25 Present Address: University of Minnesota, St. Paul, MN 55108 USA

**Keywords:** Maize, Genome, Genotype, Environment, Breeding, Phenotype, Prediction, Soil, Inbred, Hybrid

## Abstract

**Objectives:**

Crop improvement relies on analysis of phenotypic, genotypic, and environmental data. Given large, well-integrated, multi-year datasets, diverse queries can be made: Which lines perform best in hot, dry environments? Which alleles of specific genes are required for optimal performance in each environment? Such datasets also can be leveraged to *predict* cultivar performance, even in uncharacterized environments. The maize Genomes to Fields (G2F) Initiative is a multi-institutional organization of scientists working to generate and analyze such datasets from existing, publicly available inbred lines and hybrids. G2F’s genotype by environment project has released 2014 and 2015 datasets to the public, with 2016 and 2017 collected and soon to be made available.

**Data description:**

Datasets include DNA sequences; traditional phenotype descriptions, as well as detailed ear, cob, and kernel phenotypes quantified by image analysis; weather station measurements; and soil characterizations by site. Data are released as comma separated value spreadsheets accompanied by extensive README text descriptions. For genotypic and phenotypic data, both raw data and a version with outliers removed are reported. For weather data, two versions are reported: a full dataset calibrated against nearby National Weather Service sites and a second calibrated set with outliers and apparent artifacts removed.

## Objective

G2F is a multi-institutional, collaborative initiative to develop tools that efficiently predict performance of diverse maize (*Zea mays* ssp. *mays*) varieties across multiple growing conditions. G2F projects aim to collect, share, and analyze multi-year, large-scale genomic, phenotypic, and environmental datasets. The project builds on existing maize genome sequence resources by developing approaches to understand the functions of genes and specific alleles based on their expression in typical field conditions. There are many dimensions to the goal of understanding genotype-by-environment (G × E) interactions, including which genes impact which traits and trait components, how genes interact among themselves, the relevance of specific genes under different growing conditions, and how genes influence plant growth during various stages of development.

G2F projects foster integration of diverse research disciplines, including (but not limited to) genetics, genomics, plant physiology, agronomy, climatology, and crop modeling as well as analytical perspectives and tools derived from computational sciences, statistics, and engineering. Under the umbrella of G2F are enterprises such as the G × E project that began in 2014. The G × E project aims to document and measure genotypes, phenotypes, and environmental data in standard formats across more than twenty distributed field locations in North America annually. The resulting dataset is unique as it represents, to our knowledge, the most extensive publicly available dataset of its kind, reporting a consistent set of traits across common sets of fully genotyped germplasm not only across many locations, but also with relevant information reported down to the level of specific plots. Making these datasets publicly available enables researchers from many different disciplines to tackle the daunting analyses necessary to make useful predictions of crop performance. Novel data analysis approaches and tools are expected to result from the curated and organized data described here.

## Data description

Online forms were developed for logging field site coordinates, field management metadata, and other site-specific information. Datasets include:

- DNA sequences of inbreds (with and without imputation), including those inbreds used to produce featured hybrids. The process for creating files and metadata pertaining to the genotype by sequencing (GBS) process [1] is described. Data are most readily analyzed using TASSEL software [2]. Raw sequence reads generated are accessible via the Sequence Read Archive [3].
- Phenotype measurements for inbreds and hybrids. A handbook of instructions for making traditional phenotype measurements (reviewed in [4]) is available via the G2F website [5]. Traditional traits include stand count, stalk lodging, root lodging, days to anthesis, days to silking, ear height, plant height, plot weight, grain moisture, and test weight. Datatypes reported as both raw files and files with outliers removed are described in README files. Additionally, a large set of ear, cob, and kernel measurements was made with a non-traditional machine vision platform to quantify the components of yield [6]. These data are reported in millimeters with shape descriptors reported as principal components of contour data points. Cob color was reported as RGB (red/green/blue) pixel values. Kernel row number, counted manually, is reported as an integer.
- Environmental data collected by WatchDog 2700 weather stations (Spectrum Technologies) at 30-min intervals from planting through harvest. Collected information includes wind speed, direction, and gust; air temperature, dewpoint, and relative humidity; rainfall; and solar radiation. Data are reported as a calibrated set (based on calibration derived from nearby National Weather Service stations) and “clean” (based on removing obvious artifacts from the calibrated dataset).
- Soil characterizations by site (first taken in 2015) including plow depth, pH, buffered pH, organic matter, phosphorus levels (in parts per million), and potassium levels (in parts per million).

Data collected in year *n* are released to project members in spring of the following year (*n *+ *1*), and released to the public the subsequent year (*n *+ *2*). The 2014 and 2015 datasets are publicly available via the NCBI SRA [7] and CyVerse/iPlant [8] with files and access links shown in Table 1.

**Table 1:** Overview of data files and data sets

Label	Name of data file/data set	File types (extension)	Data repository and identifier
DNA Sequences of Inbreds	GBS sequencing Maize G2F (G × E) inbreds	Sequence reads	NCBI SRA PRJNA385022 [3] (https://www.ncbi.nlm.nih.gov/bioproject/PRJNA385022)
2014 Field Season Phenotypic and Genotypic Data	_readme.txt	.txt	CyVerse [9] (10.7946/P2V888)
	/a._2014_hybrid_phenotypic_data	directory	
	_g2f_2014_hybrid_data_description.txt	.txt	
	g2f_2014_hybrid_no_outliers.csv	.csv	
	g2f_2014_hybrid_raw.csv	.csv	
	/b._2014_gbs_data	directory	
	_g2f_2014_gbs_data_description.txt	.txt	
	g2f_2014_gbs_data.csv	.csv	
	g2f_2014_zeagbsv27.imp.h5	.h5	
	g2f_2014_zeagbsv27.imp.h5.gz	.gz	
	g2f_2014_zeagbsv27.raw.h5	.h5	
	g2f_2014_zeagbsv27.raw.h5.gz	.gz	
	g2f_2014_zeagbsv27impv5hmp.txt.gz	.gz	
	g2f_2014_zeagbsv27v5hmp.txt.gz	.gz	
	/c._2014_weather_data	directory	
	_g2f_2014_weather_data_description.txt	.txt	
	g2f_2014_weather_calibrated.csv	.csv	
	g2f_2014_weather_clean.csv	.csv	
	/d._2014_inbred_phenotypic_data	directory	
	_g2f_2014_inbred_data_description.txt	.txt	
	g2f_2014_inbred_no_outliers.csv	.csv	
	g2f_2014_inbred_raw.csv	.csv	
	/z._2014_supplemental_info	directory	
	g2f_2014_field_characteristics.csv	.csv	
2015 Field Season Phenotypic and Genotypic Data	_readme.txt	.txt	CyVerse [10] (10.7946/P24S31)
	/a._2015_hybrid_phenotypic_data	directory	
	_g2f_2015_hybrid_data_description.txt	.txt	
	g2f_2015_hybrid_no_outliers.csv	.csv	
	g2f_2015_hybrid_raw.csv	.csv	
	/b._2015_gbs_data	directory	
	_g2f_2014_gbs_data_description.txt	.txt	
	/c._2015_weather_data	directory	
	_g2f_2015_weather_data_description.txt	.txt	
	g2f_2015_weather_calibrated.csv	.csv	
	g2f_2015_weather_clean.csv	.csv	
	/d._2015_inbred_phenotypic_data	directory	
	_g2f_2015_inbred_data_description.txt	.txt	
	g2f_2015_inbred_raw.csv	directory	
	/e._2015_soils	directory	
	_g2f_2015_soil_data.txt	.txt	
	g2f_2015_soil_data.csv	.csv	
	/z._2015_supplemental_info	directory	
	_g2f_2015_supplemental_information.txt	.txt	
	g2f_2015_cooperator_list.csv	.csv	
	g2f_2015_field_irrigation.csv	.csv	
	g2f_2015_field_metadata.csv	.csv	
2014 and 2015 Inbred Ear Imaging	_readme.txt	txt	CyVerse [11] (10.7946/P2C34P)
	2014_2015_compiledData.tar.gz	.tar.gz	
	2014_gxe_compiledDataAndFileNames.csv	.csv	
	2014_gxe_compiledDataAndFileNames_Raw.csv	.csv	
	2015_gxe_compiledDataAndFileNames.csv	.csv	
	2015_gxe_compiledDataAndFileNames_Raw.csv	.csv	
	CEK_Data_Files.tar.gz	.tar.gz	
	/cob	directory	
	_cob.txt	txt	
	cob.tar.gz	.tar.gz	
	cob_01of05.tar.gz	.tar.gz	
	cob_02of05.tar.gz	.tar.gz	
	cob_03of05.tar.gz	.tar.gz	
	cob_04of05.tar.gz	.tar.gz	
	cob_05of05.tar.gz	.tar.gz	
	/ear	directory	
	_ear.txt	.txt	
	ear.tar.gz	tar.gz	
	ear_01of08.tar.gz	tar.gz	
	ear_02of08.tar.gz	tar.gz	
	ear_03of08.tar.gz	tar.gz	
	ear_04of08.tar.gz	tar.gz	
	ear_05of08.tar.gz	tar.gz	
	ear_06of08.tar.gz	tar.gz	
	ear_07of08.tar.gz	tar.gz	
	ear_08of08.tar.gz	tar.gz	
	/kernel	directory	
	_kernel.txt	.txt	
	kernel.tar.gz	tar.gz	
	kernel_01of05.tar.gz	tar.gz	
	kernel_02of05.tar.gz	tar.gz	
	kernel_03of05.tar.gz	tar.gz	
	kernel_04of05.tar.gz	tar.gz	
	kernel_05of05.tar.gz	tar.gz	

As technologies develop and the number of researchers involved in the project grows, it is anticipated that increasingly diverse datatypes will be documented. An example of the use of these data has been reported [12]. In that study, phenotypic plasticity was found to be disproportionately controlled by regulatory regions. Because these datasets support lines of inquiry limited only by the questions researchers pose, the potential scope of application for these data is broad. The dataset is anticipated to additionally impact the field simply by being the first public dataset of its scale that has been collected and reported using standardized protocols and formats, respectively, thus defining standards for data collection, formatting, and access.

## Limitations

Missing data occurs in most datasets. For genotypic and phenotypic datasets, missing data are left blank rather than zero or ‘null’ representation because some measured data report zero values and some software will only accept numeric values (not strings). The exception is for traits extracted from inbred ear, cob, and kernel image data, which are demarcated with ‘NA’.

In some instances, reported data were maintained rather than editing for consistency. These decisions were made to minimize misinterpretation that could lead to incorrect documentation or measurements.

For weather data, raw files reported by sensors are not provided because machine data were calibrated based on information from nearby weather stations to ensure accuracy (e.g., if the wind vane was set improperly, a calibration correction was required).

Field locations are not always identical year-to-year, primarily due to crop rotation management practices. Each field’s GPS coordinates are reported annually to enable data aggregation in keeping with specific research objectives.

Germplasm used and reported are specific to the project and are held by researchers involved in the project. They do not derive directly from national public genebanks. Seed access is granted in keeping with seed availability from cooperating researchers directly.

## Abbreviations

G2F: Genomes to Fields
G × E: genotype by environment interaction
GBS: genotyping by sequencing
RGB: red/green/blue
DOI: Digital Object Identifier
